# The Emerging Role of Protein Phosphorylation as a Critical Regulatory Mechanism Controlling Cellulose Biosynthesis

**DOI:** 10.3389/fpls.2016.00684

**Published:** 2016-05-24

**Authors:** Danielle M. Jones, Christian M. Murray, KassaDee J. Ketelaar, Joseph J. Thomas, Jose A. Villalobos, Ian S. Wallace

**Affiliations:** ^1^Department of Biochemistry and Molecular Biology, University of Nevada, Reno, RenoNV, USA; ^2^Department of Chemistry, University of Nevada, Reno, RenoNV, USA

**Keywords:** cellulose synthase, plant cell wall, protein kinases, cellulose biosynthesis, protein kinase regulation

## Abstract

Plant cell walls are extracellular matrices that surround plant cells and critically influence basic cellular processes, such as cell division and expansion. Cellulose is a major constituent of plant cell walls, and this paracrystalline polysaccharide is synthesized at the plasma membrane by a large protein complex known as the cellulose synthase complex (CSC). Recent efforts have identified numerous protein components of the CSC, but relatively little is known about regulation of cellulose biosynthesis. Numerous phosphoproteomic surveys have identified phosphorylation events in CSC associated proteins, suggesting that protein phosphorylation may represent an important regulatory control of CSC activity. In this review, we discuss the composition and dynamics of the CSC *in vivo*, the catalog of CSC phosphorylation sites that have been identified, the function of experimentally examined phosphorylation events, and potential kinases responsible for these phosphorylation events. Additionally, we discuss future directions in cellulose synthase kinase identification and functional analyses of CSC phosphorylation sites.

## Introduction

Plant cell walls are complex polysaccharide-rich extracellular matrices that surround all plant cells and critically influence basic plant cellular growth processes, such as cell expansion, cell division, and acquisition of cell shape (Somerville et al., 2004; Cosgrove, 2005). While plant cell walls are structurally heterogeneous (Pauly and Keegstra, 2008; Burton et al., 2010), cell wall polysaccharides can generally be grouped into three structural classes: cellulose, hemicellulose, and pectins. Cellulose is the most abundant component of plant cell walls and serves as the main load-bearing polysaccharide that resists the internally generated osmotic force that is required for turgor-mediated cell growth.

Cellulose is composed of extended β-(1→4)-linked glucosyl polymers and many models have proposed that these glucan chains are organized into a paracrystalline array of 18–36 chains. However, recent NMR, wide-angle X-ray diffraction scattering (WAXS), and neutron scattering studies indicate that experimental cellulose structure data is more accurately represented by models that contain 18–24 glucan chains (Newman et al., 2013; Thomas et al., 2013), suggesting that this model of cellulose structure is more realistic. Additionally, the cellulose chain varies from 300 to 10,000 glucose units (Moon et al., 2011), and the degree of cellulose microfibril crystallinity varies between plant tissues. The relative complexity and precise structure of cellulose indicates that a highly organized protein complex is necessary to catalyze cellulose biosynthesis. Additionally, the developmental and environmental stimuli that regulate cellulose biosynthesis remain largely uncharacterized. In this review, we discuss the structure and composition of the cellulose synthase complex (CSC) as well as the potential for complex cellulose biosynthesis regulation by post-translational phosphorylation. We also discuss protein kinases that have been implicated in cell wall biogenesis and suggest possible relationships to phosphorylation events in the CSC.

## Cellulose Biosynthesis and the Composition of the Cellulose Synthase Complex

While cellulose is the most abundant biopolymer on the planet, the molecular players involved in cellulose biosynthesis are not completely defined. However, numerous studies over the last decade have begun to bring the molecular composition of the CSC into focus. Early studies revealed that cellulose synthesis is catalyzed by large 25–30 nm plasma membrane complexes that associate with the termini of growing cellulose microfibrils in freeze-fracture electron microscopy micrographs (Herth, 1983; Chapman and Staehelin, 1985; Kimura et al., 1999; Bowling and Brown, 2008). These complexes exhibited six-fold symmetry and form floral-shaped structures, hence they are commonly referred to as “rosettes.” However, the protein composition of these massive protein complexes remained elusive until the last two decades.

The development and characterization of cellulose biosynthesis inhibitors (CBIs) shed light on the phenotypic defects that result from cellulose biosynthesis inhibition. Plant exposure to CBIs, such as isoxaben and dichlobenil (DCB), resulted in the inhibition of plant cell expansion, concomitant root cell swelling, and inhibition of glucose incorporation into the cellulose fraction of the cell wall (Heim et al., 1990; DeBolt et al., 2007). Subsequent genetic screens in the model plant *Arabidopsis* were performed to identify mutants that mimicked these phenotypes.

These genetic analyses revealed that the plant CSC contains three non-redundant Cellulose Synthase A (*CesA*) catalytic subunits. The *Arabidopsis* genome contains 10 *CesA* genes (Richmond and Somerville, 2000), and primary cell wall cellulose biosynthesis requires *CesA1, CesA3*, and *CesA6*-like genes (Arioli et al., 1998; Fagard et al., 2000; Desprez et al., 2007; Persson et al., 2007). *CesA1* and *CesA3* are genetically required for cellulose biosynthesis in primary cell walls, while *CesA2, 3, 5*, and *6* are partially redundant (Desprez et al., 2007; Persson et al., 2007). In *Arabidopsis, CesA4, 7*, and *8* genes are all required for secondary wall cellulose biosynthesis and mutations in these genes cause cellulose biosynthesis defects in tissues requiring secondary cell walls (Turner and Somerville, 1997). Numerous lines of evidence indicate that CESA subunits interact with one another to form higher order complexes and it is proposed that each CESA subunit synthesizes one glucan chain (Taylor et al., 2003; Atanassov et al., 2009), however, this common model should be reevaluated based on recent cellulose structural studies (Newman et al., 2013; Thomas et al., 2013). Furthermore, recent biochemical studies suggest that both the primary and secondary wall CSCs exhibit 1:1:1 subunit stoichiometry (Gonneau et al., 2014; Hill et al., 2014), and that 10–12 copies are present in each CSC (Chen et al., 2014), leading to an overall architecture of 30–36 CESA subunits per CSC.

The CSC exhibits dynamic localization and behavior in expanding interphase cells during normal growth and development. Live-cell imaging of fluorescently tagged CESA6 subunits via spinning disk microscopy revealed that CSCs are localized to the Golgi apparatus, small microtubule-associated cellular compartments (SmaCC’s/MASCs), as well as small motile puncta at the plasma membrane (Paredez et al., 2006; Crowell et al., 2009; Gutierrez et al., 2009). The PM-localized puncta are proposed to represent active CSCs at the plasma membrane, and these complexes move with an average velocity of 250 nm/min. These CSCs also move along linear trajectories that are established by cortical microtubules underlying the plasma membrane (Paredez et al., 2006; Crowell et al., 2009; Gutierrez et al., 2009). Live-cell imaging has also revealed that CBIs alter the dynamics of the CSC. For example, treatment of *Arabidopsis* seedlings with isoxaben (Paredez et al., 2006; Gutierrez et al., 2009) and other CBIs (Bischoff et al., 2009; Harris et al., 2012; Xia et al., 2014) result in the removal of CSCs from the plasma membrane. In contrast, DCB treatment prevents CSC motility and leads to increased accumulation of CSCs at the plasma membrane (DeBolt et al., 2007). These observations suggest that plasma membrane localized motile CSCs represent complexes that are actively synthesizing cellulose.

In addition to CESA subunits, genetic and transcriptional correlation analyses have identified a variety of additional accessory subunits that associate with the CSC (Brown et al., 2005; Persson et al., 2005). For example, mutations in the gene encoding the endoglucanase *KORRIGAN1* (*KOR1*) were additionally identified as cellulose biosynthesis mutants (Nicol et al., 1998; Lane et al., 2001; Sato et al., 2001). *kor1* mutants are cellulose deficient, exhibit reduced root elongation, and epidermal cell swelling phenotypes that are typical of cellulose deficient mutants. Furthermore, KOR1 was demonstrated to physically interact with CESA subunits by split-ubiquitin yeast two-hybrid, bimolecular fluorescence complementation (BiFc), and co-immunoprecipitation (Lei et al., 2014; Mansoori et al., 2014; Vain et al., 2014). Live-cell imaging studies have revealed that KOR1 co-localizes with fluorescently tagged CSCs at the plasma membrane, and moves with a similar constant velocity, indicating that this protein is a component of the CSC *in vivo.* KOR1 is an active endo-β-(1→4)-glucanase, and endoglucanase activity is required for active cellulose biosynthesis. Additionally, live-cell imaging of fluorescently labeled CESAs in *kor1* mutant backgrounds indicate that CSC velocity is reduced by 50–60%, suggesting that KOR1 is a positive regulator of cellulose biosynthesis (Paredez et al., 2008; Vain et al., 2014).

The glycosylphosphatidyl inositol (GPI)–linked protein COBRA is also genetically implicated in cellulose biosynthesis (Benfey et al., 1993; Schindelman et al., 2001). Subsequent biochemical analyses have revealed that COBRA is attached to the outer leaflet of the plasma membrane through its GPI anchor (Schindelman et al., 2001), and that this protein binds glucan chains (Liu et al., 2013), suggesting that COBRA may serve as a cellulose aggregating subunit that mediates the assembly of glucan chains.

The *Arabidopsis* genome contains 11 other *COB-Like* (*COBL*) genes, constituting a small gene family (Roudier et al., 2002). Interestingly, the transcriptional patterns of these genes vary widely, and many family members are expressed in a tissue-specific manner (Brady et al., 2007). Genetic analysis of *COBL* family members revealed that these genes play tissue-specific roles in cell wall biosynthesis. For example, the *Arabidopsis COBL10* has been implicated in pollen tube growth and guidance (Li et al., 2013), while the *COBL2* participates in cellulose biosynthesis during seed coat mucilage deposition (Ben-Tov et al., 2015). These observations suggest that COB or COBL proteins are likely associated with cellulose biosynthesis, but may be sub-functionalized to form tissue-specific cellulose structures.

Recently, two cellulose synthase interacting proteins were demonstrated to physically associate with the CSC and play important roles in cellulose biosynthesis. For example, the *Cellulose Synthase Interactive protein 1* (*CSI1*) gene was identified by yeast two-hybrid screening and co-expression analysis. CSI1 is a 2150 amino acid protein containing multiple Armadillo repeats and a C-terminal C2 domain. Deletion of the *CSI1* in *Arabidopsis* resulted in reduced cell expansion, radial cell swelling, and cellulose deficiency (Gu et al., 2010) indicating that CSI1 is indeed involved in cellulose biosynthesis. CSI1 physically interacts with both CESA subunits and cortical microtubules (Gu et al., 2010; Li et al., 2012), and live-cell simultaneous imaging of CSCs and microtubules in the *csi1* null mutant background revealed that CSI1 is required for microtubule-directed guidance of the CSC (Gu et al., 2010; Bringmann et al., 2012; Li et al., 2012). Deletion of the CSI1 C2 domain resulted in CSI1 localization to the cytosol, suggesting that this domain is important for CSC association (Bringmann et al., 2012). This domain is also essential for the association of CSI1 with microtubules (Lei et al., 2015). These observations indicate that CSI1 is required for the functional association between CSCs and cortical microtubules.

Recently, the companion of cellulose synthase (CC) proteins were identified by transcriptional co-expression analysis and demonstrated to physically interact with CESA subunits (Endler et al., 2015). The CC proteins also co-localized and co-migrated with the CSC in living cells indicating that they are also CSC accessory subunits. These proteins also physically associate with cortical microtubules, and stimulate microtubule polymerization, suggesting that the CC proteins stimulate localized microtubule assembly around the CSC.

## CSC Dynamics During Stress and Development

Various lines of evidence suggest that CSC localization and motility is highly dynamic *in vivo*. For example, the CSCs are trafficked from the *trans*-Golgi network (TGN) to SmaCC’s, which then associate with and move along cortical microtubules. These small vesicles stall occasionally on microtubules to deliver new CSCs to the plasma membrane (Crowell et al., 2009; Gutierrez et al., 2009). Once delivered to the plasma membrane, CSCs remain still for approximately 1 min before becoming motile and achieving their constant velocity (Gutierrez et al., 2009). Recent evidence also suggests that motile complexes are removed from the plasma membrane by a clathrin-mediated process involving the clathrin adapter protein AP2 (Bashline et al., 2013, 2015). These complex trafficking events conceivably necessitate signals that indicate whether the CSC should be delivered to the plasma membrane, active at the plasma membrane, or endocytosed.

Another elegant example of regulated intracellular trafficking of the CSC occurs during cell plate formation (Miart et al., 2014). During plant cell division, new cross walls must be established after nuclei have been segregated into daughter cells. An internal cell membrane termed the phragmoplast assembles at the site of the future cross wall, and cell wall biosynthetic enzymes are recruited to the phragmoplast to mediate the synthesis of new wall material. Many cellulose biosynthesis mutants exhibit incompletely formed cross walls or “cell wall stubs,” suggesting that the function of these enzymes is necessary for proper cell plate formation (Zuo et al., 2000; Lane et al., 2001). Indeed, CSCs are relocalized from the plasma membrane to the cell plate during plant cell division (Miart et al., 2014) to form new cross-walls. Fluorescently labeled CESA1, 3, and 6 were localized at the developing cell plate during cytokinesis and diffuse as the cell plate matures. Further investigation of CSC spatiotemporal dynamics during phragmoplast formation revealed that CESAs are localized to the PM as observed in normal interphase cells. After late anaphase, CESAs are recruited to the phragmoplast, and the CESA fluorescent signal gradually spreads toward the cell periphery as the cell plate grows. Finally, the CESA-associated fluorescent signal diminishes at the cell plate, which corresponds with a return of complexes localized to the plasma membrane. During this redistribution of CESAs, clathrin light chains co-localize with CESA containing vesicles (Miart et al., 2014), suggesting that this redistribution process is mediated by clathrin-dependent vesicle transport.

In some cells (e.g., xylem vascular cells), thick secondary cell wall layers are synthesized after the primary cell wall is deposited (Turner and Somerville, 1997). Cellulose is a major component of secondary cell walls and is synthesized by a unique trio of CESA proteins that are specific for secondary wall biosynthesis. In *Arabidopsis, CesA4, 7*, and *8* are responsible for secondary wall synthesis, and genetic analyses have demonstrated that disruption of these genes lead to a characteristic weakening and collapse of xylem cell walls (Turner and Somerville, 1997). Recent efforts have facilitated the *in vivo* imaging of secondary wall CESAs in living cells. While secondary CESAs continued to migrate along cortical MTs, the velocity of YFP-CESA7 CSCs was much faster than CSCs associated with primary cell wall biosynthesis. Interestingly, secondary CSC velocity changes over the course of cell differentiation with an average velocity of 290 nm/min during early development, 330 nm/min during mid-development, and 190 nm/min during late development (Watanabe et al., 2015). These observations suggest that some aspect of secondary wall CSCs make them faster (i.e., more active) than their primary wall counterparts, and that the velocity of these complexes is regulated in a temporal manner, indicating that cellulose biosynthesis is controlled by factors other than gene expression during secondary cell wall biosynthesis.

Plants are susceptible to biotic and abiotic stresses during their entire life cycle, which can lead to reduced growth and biomass production. Plant cell walls play a key role in protecting plants from harmful stresses by providing the cell with a physical barrier against the external environment. Interestingly, abiotic stress has been shown to modulate the localization of the CSC in a manner analogous to some CBIs. For example, treatment of YFP-CESA6 expressing seedlings with 200 mM mannitol induced relocalization of CSCs from the plasma membrane to SmaCCs/MASCs (Gutierrez et al., 2009), suggesting that osmotic stress leads to the inhibition of cellulose biosynthesis. Other osmotic stresses have been demonstrated to result in similar re-localization events. For example, sodium chloride treatment of dual-labeled CSC/microtubule marker lines revealed a dynamic temporal interplay between CSCs and cortical microtubules during this stress condition (Endler et al., 2015). Upon sodium chloride treatment, CSCs began to disappear from the plasma membrane after 30 min. This disappearance coincided with the depolymerization of cortical microtubules. However, after approximately 24 h, the CSCs returned to the plasma membrane following cortical microtubule repolymerization (Endler et al., 2015). These cellular dynamics upon the imposition of abiotic stress suggest that the CSC responds to regulated trafficking signals that alter localization to respond to these stresses.

Overall, these dynamic changes in CSC localization and behavior over an array of developmental and environmental conditions suggest that CSC dynamics are regulated in response to these conditions. For example, it is unlikely that CSC delivery to or internalization from the plasma membrane is simply regulated by gene expression. Similarly, it is unlikely that the re-localization responses to abiotic stress or cell division are simply regulated by changes in transcriptional events. Therefore, we hypothesize that the CSC is controlled by some other more rapid regulatory event and further suggest that post-translational modifications of the CSC could be responsible for these rapid and conditional changes in CSC dynamics.

## Proteomic Analysis of CSC Component Phosphorylation

**FIGURE 1:**
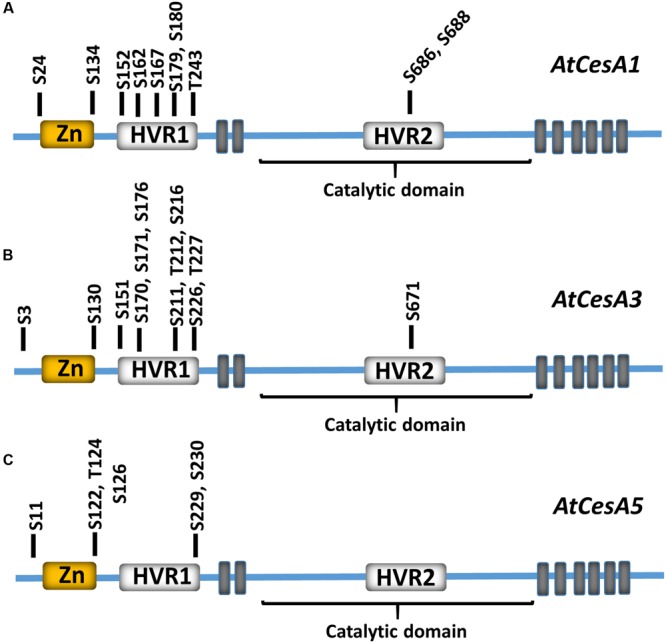
**Experimentally supported phosphorylation sites in CESA subunits.** Experimentally supported phosphorylation sites in *Arabidopsis* CESA1 **(A)**, CESA3 **(B)**, and CESA5 **(C)** are shown relative to the CESA N-terminal zinc finger (Zn; yellow), hypervariable 1 and 2 (HVR1 and HVR2; white), and catalytic domains. The residue and corresponding residue number within the primary sequence is shown. The position of predicted CESA transmembrane domains are indicated by gray boxes. These phosphorylation sites are experimentally supported in the PhosPhat 4.0 phosphorylation database.

Protein phosphorylation is one of the most widespread forms of post-translational modification in eukaryotes. The addition of a phosphoryl group to serine, threonine, and tyrosine residues by a protein kinase can regulate activity, localization, stability, and protein–protein interaction networks of phosphorylation targets. Phosphoproteomic studies have identified multiple phosphorylation sites on proteins associated with cellulose production, and these phosphorylation sites are particularly abundant in CESA proteins (Nühse et al., 2004, 2007; Taylor, 2007; Nakagami et al., 2010; Facette et al., 2013). Most CESA phosphorylation sites occur in hypervariable domains in the N-terminal domain of the CESA (**Figure 1**). These hypervariable domains show very little sequence conservation between CESA isoforms within an organism, but are highly conserved among orthologs in other organisms (Nakagami et al., 2010; Facette et al., 2013). Protein kinases typically recognize short linear amino acid epitopes surrounding the phosphorylated residue, and while the amino acids surrounding each experimentally supported CESA phosphorylation site are highly conserved (Carroll and Specht, 2011), these sequences are not conserved between phosphorylation sites. These two observations together indicate that isoform-specific regulation of CESAs through many different protein kinases is a tightly conserved feature of cellulose biosynthesis.

**FIGURE 2:**
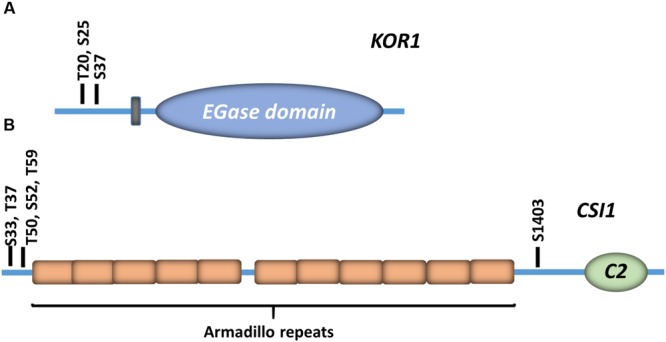
**Experimentally supported phosphorylation sites in accessory CSC subunits.** The experimentally supported phosphorylation sites in the *Arabidopsis* KORRIGAN1 (KOR1) endo-β-(1→4)-glucanase **(A)**, and Cellulose Synthase Interactive protein 1 (CSI1; **B)** are indicated. Each residue and corresponding residue indices are indicated. The predicted transmembrane domain is indicated by a gray box, and the position of the endoglucanase domain (EGase) is also shown. CSI1 Armadillo repeats are shown in orange boxes, and the position of the C-terminal C2 domain (C2) is indicated.

In addition to phosphorylation events identified in CESA proteins, various CSC-associated subunits have also been identified as phosphoproteins (**Figure 2**). For example, phosphoproteomic surveys have identified three phosphorylation sites in the cytosolic N-terminus of *Arabidopsis* KOR1 (T20, S25, and S37; **Figure 2A**). Publicly available phosphoproteomic data also indicates that CSI1 contains multiple experimentally supported phosphorylation sites within the N-terminus of the protein (**Figure 2B**). Functional analysis of CSI1 indicates that the C-terminal C2 domain is required for microtubule binding, but computational modeling further suggests that CSI1 forms a loop-shaped structure in which the N and C-terminal portions come into close contact (Lei et al., 2015). This observation suggests that phosphorylation of CSI1 may modulate intermolecular interactions within the protein to mediate microtubule binding. It should be noted that none of the sites in either KOR1 or CSI1 are in defined functional domains, nor have the functionality of any of these phosphorylation events been studied intensively. The multitude of phosphorylation events that have been identified in CSC components strongly suggest that post-translational phosphorylation is an important regulatory mechanism for the control of cellulose biosynthesis. For example, individual or multiple phosphorylation sites within these CSC components could be responsible for the observed regulated trafficking of CSCs in response to developmental or environmental cues.

## Genetic and Biochemical Analysis of Cellulose Synthase Phosphorylation

**FIGURE 3:**
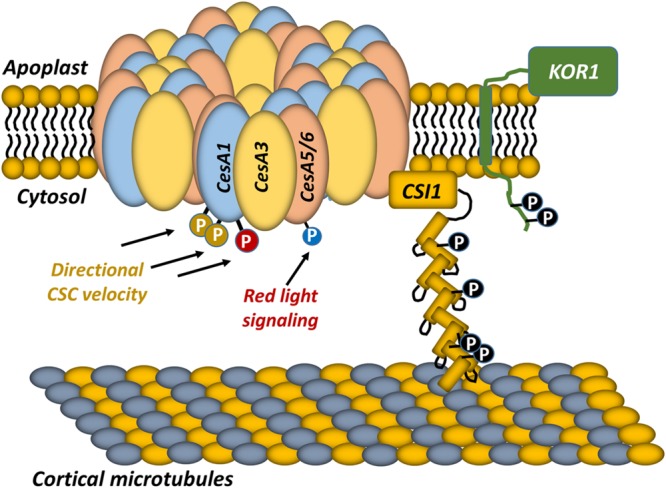
**Current model of CSC regulation by post-translational phosphorylation.** The composition of the primary cell wall CSC is shown. The CSC contains three non-redundant CESA subunits that are likely organized into a hexamer of hexamers, resulting in 36 CESA subunits within the CSC. The CSC is guided along trajectories that are defined by cortical microtubules underlying the plasma membrane, and the association of the CSC with cortical microtubules is mediated by Cellulose Synthase Interactive protein 1 (CSI1). KORRIGAN1 (KOR1) is also a component of the CSC. Each of these proteins is phosphorylated at multiple positions. Previous studies indicated that CESA5 phosphorylation (blue phosphate) is involved in regulating the velocity of the CSC during red-light stimulation (Bischoff et al., 2011). Other studies have linked CESA1 phosphorylation sites to differential CSC velocity that is microtubule-dependent (Chen et al., 2010). However, numerous phosphorylation sites within the CSC, and its accessory subunits remain to be investigated.

Several studies have attempted to address the regulatory role of CSC phosphorylation by mutating experimentally supported phosphorylation sites to phosphonull (A) or phosphomimic (D/E) residues, and observing the behavior of mutated subunits *in vivo* by imaging fluorescent CSCs (Taylor, 2007; Chen et al., 2010, 2016; Bischoff et al., 2011). For example, a recent analysis of experimentally supported phosphorylation sites in *Arabidopsis* CESA1 was performed in which each site was mutated to a phosphomimic or phosphonull residue. These phosphorylation site mutants were re-introduced into the temperature-sensitive *CesA1* mutant *rsw1-1*. Complementation analysis indicated that phosphorylation site null or mimic mutants of individual sites differentially complemented the *rsw1-1* phenotype in root or hypocotyl elongation assays as well as cellulose content assays (Chen et al., 2010), suggesting that some of these phosphorylation sites have functional implications *in vivo* under normal growth conditions. Live-cell imaging of fluorescently labeled CSCs was used to investigate the motility of these phosphorylation site modified CSCs *in vivo*. In wild-type seedlings, CSCs move at similar velocities in both directions along cortical microtubule trajectories (Paredez et al., 2006; Desprez et al., 2007; Persson et al., 2007). In contrast, CSC particle velocities were directionally asymmetric in phosphorylation site mutant lines that exhibited abnormal tissue or cell expansion (Chen et al., 2010). Upon further investigation, this directional asymmetry was dependent upon cortical microtubules because oryzalin-dependent microtubule depolymerization abolished the differential velocity effect. These observations suggest that phosphorylation of CESA1 differentially regulates interactions with microtubules and that this, in turn, alters microfibril structure in the primary cell wall (Chen et al., 2010; **Figure 3**). Recently, the S211 and T212 phosphorylation sites in CESA3 were shown to cause similar CSC velocity asymmetry. The CSC dynamics of *Arabidopsis CesA3 je5* mutants expressing *CesA3* phosphonull or phosphomimic mutations was examined, and CESA3 S211A and T212E mutants exhibited drastically different CSC velocities depending upon their direction of migration (Chen et al., 2016). Similar to CESA1 phosphorylation sites, this directional asymmetry was dependent on intact cortical microtubules, and this asymmetry was correlated to physiological defects in cell expansion. Interestingly, the CESA3 S211A and T212E mutants exhibited reduced root hair elongation, suggesting that these phosphorylation events play additional tissue-specific roles in root hair biogenesis.

In addition to these investigations of CESA1 and CESA3 protein phosphorylation, the relationship between light and cellulose synthesis was also examined in *Arabidopsis* (Bischoff et al., 2011). This investigation focused on CSC speed and revealed that the speed of CESA5-containing CSCs was significantly reduced in a *cesA*6*^prc1-1^* mutant background. However, the speed of CESA5-labeled CSCs increased in the *cesA6^prc1^*^-^*^1^* background after red-light pretreatment (Bischoff et al., 2011). In plants, red and far-red light perception is mediated by phytochromes, which are an ancient class of protein kinase that respond to these light signals (Quail, 2002). Interestingly, four phosphorylation sites in the N-terminal domain of CESA5 have been experimentally observed in previous phosphoproteomic surveys (Nühse et al., 2004). Mutation of all four of these experimentally supported phosphorylation sites in the CESA5 N-terminus to phosphomimic residues resulted in an increase in CESA5 speed in the absence of red-light pretreatment in the *cesa6^prc1^*^-^*^1^* background, linking the phosphorylation of CESA5 to Phytochrome B (PHYB) signaling and increased cellulose biosynthesis (Bischoff et al., 2011; **Figure 3**). This analysis of *CesA5* mutants revealed a direct relationship between CESA5 speed and PHYB signaling, although it was not demonstrated that PHYB directly phosphorylates CESA5. Due to the fact that PHYB migrates to the nucleus during red-light treatment (Matsushita et al., 2003; Chen et al., 2005), it is unlikely that this kinase directly phosphorylates CESA5, suggesting that other PHYB regulated genes are responsible for the observed increase in CSC velocities. Interestingly, combined metabolomic and proteomic studies have demonstrated that cellulose biosynthesis is modulated in response to light and CO_2_ availability, with the highest rates of cellulose synthesis occurring during low CO_2_ conditions in the light, and very little comparative cellulose biosynthesis occurring in the dark (Boex-Fontvieille et al., 2014). While this result agrees with the increased activity of CESA5 in response to red light treatment, quantitative phosphoproteomics experiments indicate that CESA5 was phosphorylated in the dark at two novel sites (S124 and S126), and that these sites were upregulated under dark conditions (Boex-Fontvieille et al., 2014). It is necessary to note here that all CESA5 phosphorylation site mutants in the original PHYB study (Bischoff et al., 2011) were mutagenized to either phosphomimic or phosphonull residues simultaneously. Therefore, these contradictory results could be due to fine regulation of CESA5 speed in the presence or absence of light.

Phosphorylation of secondary CESA proteins has also been observed. Using affinity purification and mass spectrometry, CESA4 and CESA7 were also shown to be phosphorylated in their N-terminal domains within the hypervariable region, similar to that of phosphorylation events directly observed in primary cell wall CESAs. For example, CESA7 *in vivo* phosphorylation sites were identified in wild-type plants in the hypervariable regions of *Arabidopsis* CESA4 and CESA7 (Taylor, 2007). Upon further investigation, recombinant N-terminal fragments of CESA7 revealed that phosphorylation influences the stability of the CESA7 protein and that co-incubation with the proteasome inhibitor MG132 stabilizes both N-terminal fragments and endogenous CESA7. While the functional implications of these phosphorylation sites remain to be determined *in vivo*, these findings suggest that phosphorylation of CESA7 may regulate the stability of the secondary CSCs, and these results agree with investigations of the secondary CSC in living cells (Taylor, 2007). Additional secondary CESA phosphorylation sites have been recently identified in maize (Facette et al., 2013), and these sites are conserved in *Arabidopsis* secondary wall CESAs, so it will be important to examine the functional relevance of these phosphorylation events, and their interplay with known phosphorylation events *in vivo*.

While numerous phosphorylation sites have been identified in CSC components, and these preliminary studies highlight the functional relevance of these phosphorylation sites *in vivo* and *in vitro*, it is important to consider that no protein kinase has been demonstrated to directly phosphorylate an experimentally supported CSC phosphorylation site. Identification of cognate CSC protein kinases will be an important future step in the investigation of cellulose biosynthesis regulation, and this idea is discussed further in the conclusion of this manuscript.

## Regulation Of Bacterial Cellulose Synthesis: A Potential Structural Paradigm for the Regulation of Plant Cellulose Biosynthesis By Phosphorylation

Certain species of bacteria produce complex extracellular polysaccharides that mediate biofilm formation. Among these species, some, including *Gluconacetobacter xylinus*, produce a β-(1→4)-linked paracrystalline polysaccharide resembling plant cellulose. Genetic screens in this bacterial species have revealed that several genes in the bacterial cellulose synthase (*bcs*) operon are fundamentally required for bacterial cellulose biosynthesis (Wong et al., 1990). These genes include *bscA, B, C, D*, and *Z* (Römling and Galperin, 2015). The bacterial synthase A (*bcsA*) is a homolog of the eukaryotic *CesA* gene family. In *Rhodobacter sphaeroides, in vitro* studies have shown the *bcsA* and *bcsB* are both genetically required for active synthesis of cellulose (Standal et al., 1994). In addition, purification of BcsA and BcsB subunits individually and recombining *in vitro* does not restore catalytic activity, suggesting both need to be transported to the membrane at the same time to generate a functional complex (Omadjela et al., 2013). Recently, the structure of the BcsA/BcsB complex was elucidated by x-ray crystallography at 3.5 Å resolution (Morgan et al., 2013). This structure has given insight into the mechanism of bacterial cellulose synthesis and regulation.

The crystallized BcsA-BcsB complex forms a heterodimer that is anchored in the membrane by nine transmembrane (TM) domains (eight from BcsA and one from BcsB). The eight BcsA TM domains form an open pore that is connected to the BcsA glycosyltransferase (GT) domain on the cytosolic side of the membrane, and the BcsB domain on the extracellular face of the membrane. Three interfacial amphipathic protein segments form this interface between the BcsA TM-domains and the GT domain.

The BcsA-BcsB heterodimer was crystallized in the presence of a UDP molecule and an emerging glucan chain, facilitating a more complete understanding of the bacterial cellulose synthase mechanism (Morgan et al., 2013). A single UDP molecule is bound at the bottom of the GT domain. The emerging β-(1→4)-linked glucan chain is oriented above the UDP molecule and extends through the GT domain active site through a pore formed by the TM domain, and into the extracellular space, where this glucan interacts with the BcsB domain (Morgan et al., 2013). This architecture suggests that each BcsA monomer synthesizes an individual glucan chain and that single UDP-glucose molecules are processively bound, and glucose residues are added one-by-one to the emerging glucan chain. Recent *in crystallo* enzymology experiments have confirmed this proposed processive mechanism (Morgan et al., 2016).

The bacterial BcsA cellulose synthase is directly regulated by cyclic-di-GMP (c-di-GMP; Cotter and Stibitz, 2007). C-di-GMP is composed of two GMP molecules covalently bound at the 5′ and 3′ positions by phosphodiester bonds. This molecule is accepted as a universal bacterial second messenger, known to regulate cell-cycle, virulence, and a number of other bacterial processes (Römling et al., 2013). BcsA is activated by c-di-GMP through binding of this small molecule to the PilZ domain located at the C-terminus of BcsA, where the conserved RXXXR (X represents any amino acid creating a flexible linker domain) motif directly interacts with c-di-GMP. The binding of c-di-GMP facilitates conformational changes in a flexible gating loop connecting the third interfacial domain to the remainder of the GT domain, moving the gating loop to hydrophobically interact with the second interfacial helix (IF). This interaction opens the GT domain active site and allows UDP-glucose to enter allowing cellulose synthesis to proceed (Morgan et al., 2014, 2016).

The regulation of the BcsA/BcsB may provide clues into the structural regulation of plant CESAs. Structural analysis of the BscA/BcsB complex revealed that a critical salt bridge between R580 and E371 stabilized the inactive conformation of BcsA, and the substitution of BcsA Arg580 to alanine increased BcsA catalytic activity in the absence of c-di-GMP (Morgan et al., 2014). Phosphorylation events often create new charge–charge interactions in protein domains, so it is possible that similar conformational changes lead to changes in plant CESA activity upon CESA phosphorylation. In light of this hypothesis, it is important to note that many phosphorylation sites have been identified in the proposed catalytic domain of plant CESAs near the region homologous to the BcsA gating loop, suggesting that phosphorylation of this region could alter the properties of CESA enzymatic activity.

## Protein Kinases Regulating Cellulose Deposition

A large number of phosphorylation sites in CSC components have been identified, but protein kinases mediating these phosphorylation events have not been discovered. Plant genomes typically contain a large diversity of protein kinase genes, and the *Arabidopsis* genome is predicted to contain approximately 1000 protein kinase genes (Shiu and Bleecker, 2001; Cheng et al., 2002; Champion et al., 2004). This combinatorial problem makes it challenging to identify protein kinases responsible for each experimentally identified phosphorylation event in the CSC. However, genetic analyses in *Arabidopsis* have identified a handful of protein kinase genes that play a role in cell wall biosynthesis and may therefore be good candidates for CSC protein kinases. Based on the number of unique CSC phosphorylation sites (Nühse et al., 2004, 2007; Nakagami et al., 2010; Facette et al., 2013), many protein kinases may play a role in CSC regulation.

Genetic analyses have identified a number of receptor-like kinases that are implicated in cell wall biosynthesis, which can be divided into sub-families including; the Ser/Thr RLKs, the *Catharanthus roseus* RLK1-Like (CrRLK1L) kinases, the wall-associated kinases (WAKs), and the leucine-rich repeat (LRR RLKs) kinases. For example, the THESEUS1 RLK, a member of the CrRLK1L kinases, was genetically identified as a suppressor of the *PROCUSTE1 CesA6* mutation (Hematy et al., 2007). The *prc1-1* mutation in *CesA6* results in observed growth inhibition and ectopic lignin deposition in *Arabidopsis* cells (Fagard et al., 2000). Genetic analyses revealed that the *the1* mutant partially suppressed the *prc1-1* mutant, suggesting that THE1 was partially responsible for the growth defects observed in *prc1-1*. FTIR, cellulose content and biochemical analyses of cellulose biosynthesis revealed that the *the1-1/prc1-1* double mutant is a cellulose deficient mutant, and cellulose biosynthesis was not restored in the *the1-1/prc1-1* mutant. To confirm the kinase activity of THE1, *in vitro* studies were conducted on the purified THE1 protein kinase domain which demonstrated an active kinase domain in the C terminus of the protein, which is consistent with the previously characterized data for other CrRLK1 kinases (Schulze-Muth et al., 1996; Hematy et al., 2007). Through fluorescence microscopy and promoter::GUS expression analyses, THE1 was localized to the PM in expanding cells and vascular tissue, indicating a functional role in cell growth. Overexpression of *THE1* in a WT background displayed no phenotypic variation from typical WT growth, suggesting THE1 is activated through the suppression of CSC activity. Overexpression lines of *THE1* in *eli1-1* and *pom1-2* backgrounds resulted in growth inhibition and the enhanced accumulation of lignin, as compared to the original mutant backgrounds alone. These observations suggest that the phenotypic changes characteristic of CSC mutants is in part due to the suppression of cell expansion and enhanced lignin accumulation mediated by THE1. Based on the observation that THE1 is an active protein kinase, these results also potentially suggest that THE1 may phosphorylate either direct (i.e., CSC components) or indirect downstream targets that respond to cellulose biosynthesis inhibition and mediate cell wall integrity sensing. Components of the CSC could represent THE1 phosphorylation targets based on the large number of phosphorylation events in the CSC components and plasma membrane co-localization of THE1 and the CSC (Hematy et al., 2007).

The CrRLK1L kinases have also been broadly demonstrated to coordinate cell growth, cell–cell communication, and cell wall remodeling. For example, ANXUR1 and ANXUR2 were demonstrated to play a role in the maintenance of pollen tube cell wall integrity (Boisson-Dernier et al., 2009, 2013). Over-expression of *ANXUR1* or *ANXUR2* led to growth inhibition and rupture of pollen tubes. Subsequent analyses concluded that pollen tube lengths of *ANX* overexpressing mutants (*ANX^OE^*) were significantly reduced in comparison to WT and *ANX* complement lines. These pollen tube defects in *ANX^OE^* mutants led to decreased transmission of male gametophytes and male sterility (Boisson-Dernier et al., 2013). It was concluded that the reduced pollen tube elongation and subsequent pollen tube bursting phenotypes in *ANX^OE^* lines was not due to reduced cellulose biosynthesis, but instead, resulted from imbalanced endocytosis vs. exocytosis. Based on FRAP analysis, it was evident that the recovery rate for the *ANX^OE^* mutants was significantly faster than controls, suggesting that exocytic vesicle delivery to the pollen tube tip outpaced vesicle removal. This situation could lead to overaccumulation of cell wall polysaccharides including cellulose, at the pollen tube tip, therefore restricting expansion and causing plasma membrane invagination (Boisson-Dernier et al., 2013).

The *FEI1* and *FEI2* LRR RLK subfamily members have also been genetically implicated in cellulose biosynthesis. Single *fei1* and *fei2* insertional mutants do not exhibit growth defects under normal growth conditions, but root cell expansion in *fei1fei2* double mutants was drastically reduced when these mutants were grown on media containing 4% sucrose, which facilitates maximal cell expansion rates and the identification of cell wall mutant defects. Additionally, the *fei1fei2* double mutant exhibited radial cell swelling and cellulose deficiency, suggesting that these kinases play some role in cellulose biosynthesis (Xu et al., 2008). Additional studies assessing the activity of *FEI1* and *FEI2* have demonstrated non-redundant kinase functions for these genes in seed coat mucilage cellulose biosynthesis. The *fei2* mutant demonstrated significantly reduced seed coat cellulose microfibril array deposition accounting for the altered seed coat mucilage adhesion in this mutant. Genetic complementation was used to assess the non-redundant functionality of *FEI1* and *FEI2* observed by the *fei1* and *fei2* phenotypes. *FEI2* was demonstrated to rescue the mutant phenotype of *fei1fei2* and *fei2* to that of the WT phenotype, suggesting that the non-redundant function of the *FEI* genes is a result of the distinct function of *FEI2* in cellulose deposition in seed coat mucilage (Harpaz-Saad et al., 2012).

## Conclusion and Future Perspectives

While the composition of the CSC continues to be elucidated, an understanding of CSC regulation is still in its infancy. It is clear that multiple components of the CSC are post-translationally modified by protein phosphorylation (**Figure 3**), and that some of these phosphorylation events physiologically influence the behavior of the CSC (Taylor, 2007; Chen et al., 2010, 2016; Bischoff et al., 2011). However, numerous phosphorylation events in CSC components remain to be investigated, and the protein kinases and upstream stimuli that mediate each experimentally supported CSC phosphorylation event remain to be identified.

Functional evaluation of individual CSC phosphorylation sites is an important aspect of understanding CSC function, and these experiments are arguably underway based on previous studies (Taylor, 2007; Chen et al., 2010; Bischoff et al., 2011). However, it is increasingly important to identify the protein kinases responsible for CSC regulation. This is a complex and combinatorial problem because most plant genomes contain over 1000 protein kinases, and numerous experimentally supported phosphorylation sites exist in the CSC. We highlight that numerous techniques have been developed to establish plant protein kinase-substrate relationships, and that these techniques would be extremely beneficial if applied to the CSC system. For example, the kinase-client (KiC) assay has been developed to establish rapid identification of protein kinase-substrate interactions through a mass spectrometry-based approach (Huang and Thelen, 2012; Ahsan et al., 2013). Additionally, peptide and protein microarrays have been successfully utilized to identify protein kinase substrates of plant phosphoproteins (Popescu et al., 2009; Ma and Dinesh-Kumar, 2015). It is conceivable that a large number of CSC-derived peptides could be screened via these assays against a broad range of genetically implicated kinases associated with cellulose biosynthesis to identify potential kinase-substrate relationships. Identifying these kinases may lead to a better understanding of the upstream developmental and environmental conditions regulating cellulose biosynthesis.

In the future, it will be important to understand these phosphorylation events in more detail. From a plant biotechnology perspective, it is important to understand the regulatory controls that influence cellulose biosynthesis in the interest of developing plant varieties that consistently produce more cellulose for biofuel applications. Additionally, it is important to understand how cellulose biosynthesis is regulated from a plant developmental standpoint, due to the critical role that plant cell wall biosynthesis plays in plant growth. A further understanding of the relationship between CSC phosphorylation, the protein kinases that catalyze these phosphorylation events, and the upstream stimuli that activate these phosphorylation events will, therefore, greatly enhance our understanding of plant cell wall biosynthesis.

## Author Contributions

All authors listed, have made substantial, direct and intellectual contribution to the work, and approved it for publication.

## Conflict of Interest Statement

The authors declare that the research was conducted in the absence of any commercial or financial relationships that could be construed as a potential conflict of interest.
